# Landscape use by large grazers in a grassland is restructured by wildfire

**DOI:** 10.1371/journal.pone.0297290

**Published:** 2024-02-13

**Authors:** Aishwarya Subramanian, Rachel M. Germain

**Affiliations:** 1 Department of Biology, Irving K. Barber Faculty of Science, University of British Columbia Okanagan, Kelowna, BC, Canada; 2 Department of Zoology, University of British Columbia, Vancouver, BC, Canada; 3 Biodiversity Research Centre, University of British Columbia, Vancouver, BC, Canada; Bowling Green State University, UNITED STATES

## Abstract

Animals navigate landscapes based on perceived risks vs. rewards, as inferred from features of the landscape. In the wild, knowing how strongly animal movement is directed by landscape features is difficult to ascertain but widespread disturbances such as wildfires can serve as natural experiments. We tested the hypothesis that wildfires homogenize the risk/reward landscape, causing movement to become less directed, given that fires reduce landscape complexity as habitat structures (e.g., tree cover, dense brush) are burned. We used satellite imagery of a research reserve in Northern California to count and categorize paths made primarily by mule deer (*Odocoileus hemionus*) in grasslands. Specifically, we compared pre-wildfire (August 2014) and post-wildfire (September 2018) image history layers among locations that were or were not impacted by wildfire (i.e., a Before/After Control/Impact design). Wildfire significantly altered spatial patterns of deer movement: more new paths were gained and more old paths were lost in areas of the reserve that were impacted by wildfire; movement patterns became less directed in response to fire, suggesting that the risk/reward landscape became more homogenous, as hypothesized. We found evidence to suggest that wildfire affects deer populations at spatial scales beyond their scale of direct impact and raises the interesting possibility that deer perceive risks and rewards at different spatial scales. In conclusion, our study provides an example of how animals integrate spatial information from the environment to make movement decisions, setting the stage for future work on the broader ecological implications for populations, communities, and ecosystems, an emerging interest in ecology.

## Introduction

Movement is a necessity of life for many species despite its inherent risks. Moving exposes individuals to the possibility of injury, predation, or starvation (risks; [1]), but in its absence, individuals would be deprived of essential resources, such as food, shelter, and mates (rewards; [2, 3]). To tip this balance, animals make movement decisions based on perceived risks vs. rewards, using cues from their environments that may or may not align with actual risks vs. rewards [4]. For example, actual risk might vary with the activity, distribution, and hunting mode of predators (including humans [5, 6]), whereas perceived risks may be based on correlates of actual risk [7–9], such as landscape features that tend to house or conceal predators or impede hiding or escape (i.e., the ‘evasion landscape’ [6]). Importantly, in environments where risks and rewards are perceived to vary spatially, movement may become directed towards certain locations and away from others, what we will refer to here as ‘directed movement’ for brevity—for example, it is common to find deer paths weaving through landscapes [10, 11], formed by repeated trampling along specific routes. Concentrating activities in familiar areas, such as those that would cause paths to form, is thought to be a strategy for animals to reduce uncertainty as they navigate through their environments [12, 13].

Decades of studies have demonstrated that risks and rewards affect animal behaviour in different structural environments in controlled lab conditions (e.g., [14, 15]), and in the wild, the directedness of movement is an emerging interest [16, 17] given its relevance to the structure and functioning of ecosystems [18–22]. One way to understand these connections is to conceptualize a landscape as a network of paths, representing the realization or lack thereof (if absent) of movement. Paths that are well used between some locations relative to others reflect movement that is highly directed. Although a high degree of directedness might imply that there is a constricted set of travel pathways an animal must follow, here, we instead imagine that paths connecting the landscape are self-organized by animals based on decisions made to improve survival. If examined over time, for example, in response to some environmental impact [23], temporal changes in network structure can arise as paths are gained and lost, an outcome of behavioural plasticity [24].

**Fig 1:**
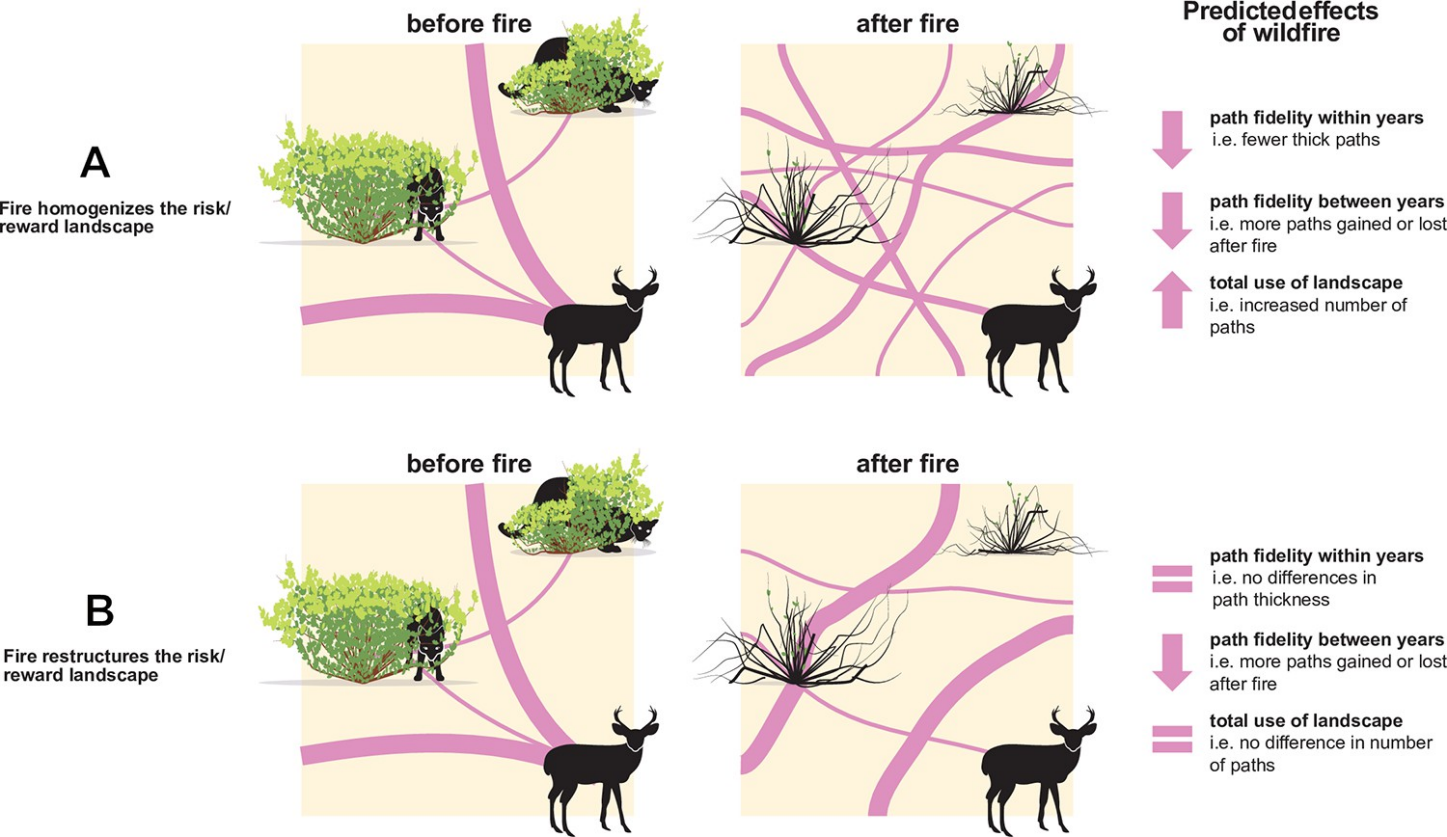
Predicted effects of wildfire on deer movement in support of alternative hypotheses. Effects include a change in the path fidelity within years (i.e., their thickness, a proxy for the directedness of movement), changes in path fidelity between years (i.e., the persistence of paths between years), and overall activity (i.e., total number of paths, with or without weighting by path thickness). All predictions are relative to sites with no recent history of fire to control for temporal changes in landscape use by deer unrelated to wildfire (a feature of Before/After Control/Impact (’BACI’) experimental designs). If wildfire has no effect on the spatial structure of risks and rewards, then we would expect no difference before vs after the fire. Equal sign = no difference between burned and unburned. Artwork is original and created by S. Heredia (https://www.sylviaheredia.com/).

Wildfires are natural experiments for examining causes of directed movement in natural landscapes [25]. In grassland and shrubland ecosystems, as wildfires pass through landscapes, they burn tall shrubs and trees that add structure to habitats [26–28], resulting in a several-years period of reduced visual and physical obstructions until vegetation recovers. For certain prey species, like those hunted by stalking predators, fewer obstructions lowers and homogenizes the risk environment: it is easier for prey to see and escape predators, affecting behaviour. For example, deer present in high-visibility landscapes like grasslands spend more time foraging, as there are less time demands on vigilance against stalking predators [29]. As a consequence, one might also expect that movement would become less directed as the risk environment becomes more homogeneous, resulting in a more even use of habitat (i.e., increased loss of old well-used paths and gain of many new lightly-used ones; Fig 1A), however, whether or not this is true depends on rewards. A common outcome of wildfires is the “magnet effect”, where large grazers are drawn to burned areas as nitrogen released from woody plants increases non-woody forage [30]. If rewards vary spatially, for example, in a nutrient shadow around burned shrubs or if different areas within an animal’s home range are differentially impacted by fire, movement in landscapes may instead be redistributed (i.e., increased loss of well-used old paths and gain of new well-used ones; Fig 1B) as opposed to homogenized by wildfire. A redistribution of movement may also occur if the spatial distribution of rewards is unaffected by disturbances but can be accessed more easily or with fewer risks.

Here, we explored whether animal movement in a landscape is rewired by wildfire, analysing a history of paths formed by primarily by mule deer (*Odocoileus hemionus*) in grassland habitat in pre-wildfire and post-wildfire years. At our particular study site in Northern California, wildfires have increased in frequency and severity to unprecedented levels. In 2020 alone, California experienced six of the 20 largest wildfires the state has ever experienced, burning a record-setting ~12,500 km^2^. This study system is ideal for three reasons. First, wildfires in 2015 impacted approximately half of our study site (see map and photos in S1 Fig), allowing comparisons between areas that were or were not impacted by wildfire. Second, tall shrubs and trees that surround and punctuate grassland habitat affect the visual environment, concealing large predators, but are burned by wildfire. Third, deer paths were numerous and clearly visible in satellite imagery of grassland habitat. We used data collected on deer paths to test the hypothesis that wildfire affects deer movement in ways that would be expected given a reduction in the complexity of the visual environment (Fig 1).

## Methods

### Study site

Our study was conducted using remote satellite imagery of the Donald and Sylvia McLaughlin Natural Reserve, at the boundaries of Lake, Napa, and Yolo counties in Northern California, USA (38.8712° N, 122.4193° W). The reserve spans an area of 28.53 km^2^ and is primarily composed of annual grassland, oak woodlands, and chaparral. Mule deer are the most abundant large mammals at the reserve, with common predators including coyotes, mountain lions, bobcats, grey foxes, and black bears. The reserve is of a Mediterranean climate, with cool wet winters and hot dry summers, and supports a fire-adapted ecosystem, with fires increasing in frequency in recent decades with human activity [31]. Two prominent wildfires impacted ~half the reserve over a time interval relevant to our study: the Rocky Fire in July 2015 and the Jerusalem Fire in August 2015, that merged at the Reserve and burned a total of ~370 km^2^ (S1 Fig). Both fires were caused by arson. Prior to 2015, the reserve had not substantially burned in recent history, other than localized impacts to the reserve’s east-end boundary in 1999 and 2004.

We focused our study on areas of the reserve that are primarily composed of annual grassland habitat, including areas punctuated or surrounded by tall shrubs or trees, for two reasons. First, logistically, deer paths in grassland are easily visible in satellite imagery, which would be obscured in more covered environments. Second, our hypotheses pertain to the structural complexity of an ecosystem, given that habitat complexity can affect risk perception by animals (e.g., [4, 32]). Fire impacts the structural complexity of different types of habitat, as perceived by large mammals, to different degrees. Most dramatically, chaparral and oak woodlands experience reductions or losses of aboveground structure, which regenerates through stored roots or a seed bank to reach maturity over 10+ years [33]. At maturity, chaparral vegetation stands 1 to 2 m tall with a thick crown that is difficult to move or see through. At the reserve, we previously found that signs of predators (e.g., carcasses, scat) were concentrated under tall trees and shrubs (pers. obs. during scat survey [21]). In contrast to the trees and shrubs that punctuate the grasslands, the grassy regions themselves regenerate in the growing season immediately following the fire [34], meaning that the loss of trees and shrubs likely constitute the largest change to the physical structure of the habitat as experienced by large mammals. Because of this regeneration and the fact that we sampled satellite imagery three years after the fire, we assume that grass in burnt and unburnt sections of grassland are similarly susceptible to trampling.

### Experimental design and data collection

We used Google Earth Pro v. 7.3.3.7786 to access satellite imagery of the McLaughlin Natural Reserve. By examining available historical layers, we chose two layers: one from a pre-wildfire year (August 2014) and one from a post-wildfire year (September 2018). Satellite imagery for the months between August 2014 and September 2018 were of poor quality, where deer paths were either undistinguishable or unidentifiable, hence we did not examine additional historical layers. Notably, this method was inspired by observing deer paths from satellite imagery for a previous project [11] and needing to pivot our research for remote work during the COVID-19 pandemic.

Using those two historical layers, we set up a sampling scheme designed to disentangle the effects of wildfire on deer movement from temporal trends unrelated to wildfire, specifically, a Before/After Control/Impact (BACI) design. In Google Earth, we delineated areas of the reserve that were impacted by wildfire in 2015 from areas that had no recent history of wildfire. Within each type of area, we selected nine square 33,405 m^2^ plots, placed randomly; within these plots, the 2015 fires caused on average a 63% loss of tree or shrub cover (compared to a 5% gain between 2014 and 2018 in unburned plots; see S2 Fig and S1 File). Plots in the burnt areas were 0.9 to 7.1 km from the fire perimeter, whereas plots in unburnt areas were 0.2 to 5.3 km (plots were evenly spread within these ranges; see map in S1 Fig). Due to the large area covered by each plot, counting every deer path would have been infeasible. Hence, within each plot, we placed five square subplots within which deer paths were enumerated. Each subplot covered 1,393 m^2^, placed randomly within each plot except with the criterion that no more than 25% of the subplot’s area was occupied by shrubs, as shrubs obstructed our ability to measure deer paths. The subplots were ~37 m wide, or ~18.5 m to a subplot’s edge if standing in the subplot’s centre, which aligns with the limited visual acuity and spatial resolution (compared to humans) reported for several deer species (e.g., white-tailed deer [35]); poor vision might in-part explain why path-forming behaviours are common in deer.

We enumerated every visible deer path in every subplot, in each of the two sampling timepoints (August 2014 and September 2018, described above). Each path was categorized based on thickness: thick, medium, or thin. Path thickness serves as a measure of repeated use, with fewer, thicker paths expected if movement patterns are directed by landscape characteristics [36]. Three thickness categories were decided upon after an initial training period, as three categories captured sufficient variation in thicknesses observed across plots. Roughly, thick paths were twice as wide as medium paths and four times as wide as thin paths (S3 Fig). Due to the potential subjectivity of categorization, only one person (A. Subramanian) recorded data on path thickness to maintain consistency across all images; this measurement subjectivity is not unique to our study, for example, in plant ecology where visually estimating percent cover is common practice. Of course, visible deer paths are not a perfect measure of deer movement (e.g., if movement is so undirected that no paths form), however, directedness should still be quantifiable via relative differences in how paths of different thicknesses respond to wildfire (e.g., an increase in thin paths likely also corresponds to an increase in paths so thin that they are not visible).

Additionally, for every subplot, we examined every deer path across the two sampling timepoints to identify if a path was unique to one time point or if it was observed in both time points. The loss of old paths and the formation of new paths in 2018 provides an estimate of how deer movement has been redistributed in a landscape, which may be expected more in landscapes that have undergone structural changes [36, 37]. We will refer to the persistence of paths across both years as ‘path fidelity between years’, whereas we will refer to the repeated use of paths within a year (our measure of ‘directedness’) as ‘path fidelity within years’. Certain rules were followed when measuring path occurrences: (1) each path was only counted once; (2) if a path from 2014 was observed in 2018 but differed in length, either longer or shorter, it was considered the same path and only counted once; (3) if a path from 2014 was observed in 2018 but differed in thickness, either thicker or thinner, it was considered the same path and only counted once. Given that each path in each year was categorized by thickness, we used a contingency table to record path transitions through time: the loss or gain of certain sizes or changes in size (S3 Fig), as all of these transitions reflect the spatial redistribution of movement in a landscape.

### Statistical analyses

All analyses were performed using R v. 4.0.3 (R Core Team, Vienna, Austria, 2019) and all mixed models were performed using the package ‘glmmTMB’ [38]. For all analyses, we used generalized linear mixed models (glmm) with the number of paths as response variables and ‘plot id’ as a random factor to account for multiple subplots per plot [39]. Because the number of paths is a count variable and can be zero-inflated, we repeated all models using Poisson, type I negative binomial, and type II negative binomial error distributions, with or without zero inflation. We then used the R package DHARMa (i.e., a package that runs diagnostics on hierarchical models) to select the single model that was most appropriate for each dataset.

We used the general approach above to test our predictions for three response variables under alternative hypotheses (Fig 1). Prediction 1: To test our prediction that path fidelity between years is affected by wildfire, we performed a glmm with the following structure: number of paths observed ~ year(s) of observation (i.e., 2014 only, 2018 only, or in both years) × plot type (i.e., impacted or not impacted by fire) + (1|plot id). Prediction 2: To test if path fidelity within years (i.e., a measure of ‘directedness’ of movement) is affected by wildfire, we performed a glmm with the following structure: the number of paths in each thickness category (i.e., one observation per thickness category per subplot per year) ~ year × path thickness × plot type (i.e., impacted or not impacted by fire) + (1|plot id/subplot id). ‘Subplot id’ was included as a random factor given that there were three thickness measures within each subplot. Prediction 3: Lastly, to understand if the overall amount of movement activity was affected by fire, we performed two analyses: one weighted by path thickness and one that was unweighted. Specifically, because paths are thicker because they are used more by deer, we multiplied the path counts in each subplot by the following weights: thin = 1, medium = 2, thick = 4. These weightings reflect relative size differences compared to thin paths. We then summed together the weighted or unweighted counts to get a single path count per subplot, which were used in a model with the following structure: year × plot type (i.e., impacted or not impacted by fire) + (1|plot id).

### Ethics statement

No live vertebrates were used in this study; all movement data was extracted remotely from satellite imagery. Thus, no permitting was necessary.

## Results

**Fig 2:**
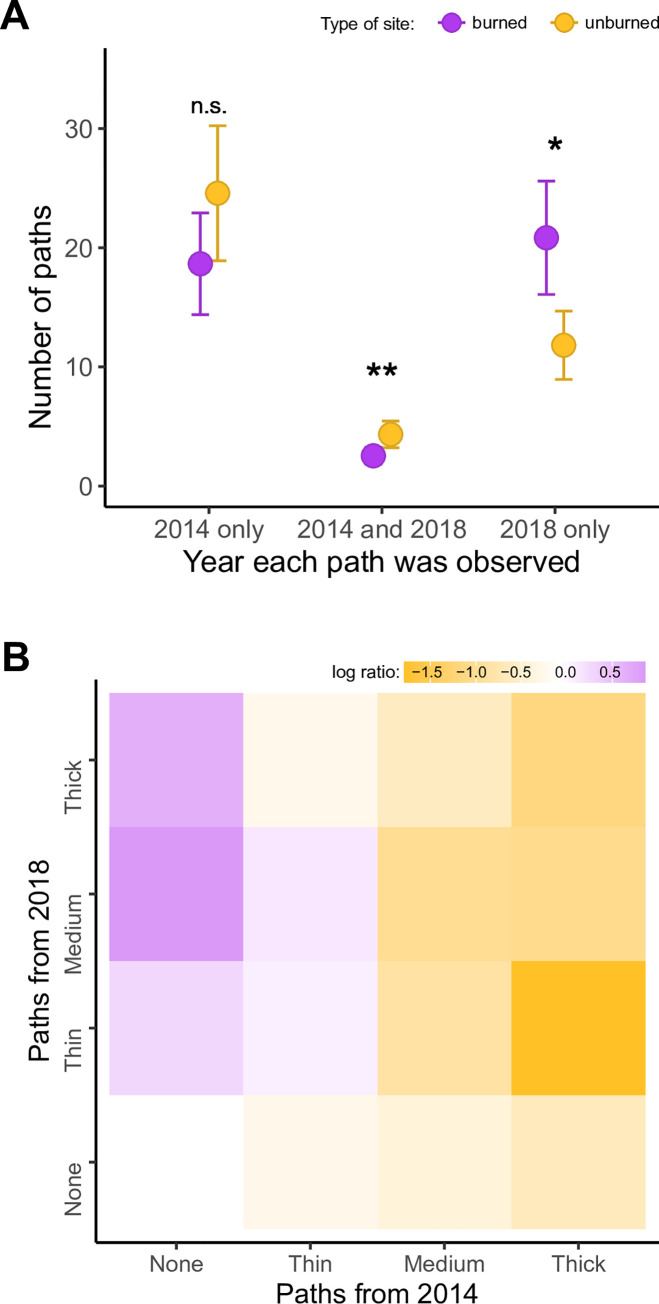
Deer movement is restructured by wildfire. (A) More new paths were formed (seen in 2018 only) and fewer old paths persisted (seen in both 2014 and 2018) in burned (magenta points) compared to unburned (yellow points) areas. Points and error bars are fitted means and 95% confidence intervals from glmm; *P < 0.05, **P < 0.01, n.s. *P* > 0.05. (B) Paths appeared (first column), disappeared (first row), remained the same (diagonal) between sampling years, or transitioned in size (all other cells) more often in burned (magenta cells) or unburned (yellow cells) areas. Cell colors/shading are log ratios of burned/unburned path counts in each size category. Data on burned and unburned sites is presented in S4 Fig.

As predicted, burned sites exhibited lower path fidelity within years and lower path fidelity between years (i.e., persistence of paths through time) than unburned sites across the two years (i.e., significant interaction between path occurrence x plot type (*χ*^2^ = 39.3, *P* < 0.001); Fig 2A). Interestingly, lower path fidelity within years was primarily achieved by gains of new paths (i.e., ‘2018 only’ in Fig 2A, *χ*^2^ = 6.2, *P* = 0.013) and not the loss of old paths (i.e., ‘2014 only’ in Fig 2A, *χ*^2^ = 1.5, *P* = 0.215). By examining thickness classifications of paths that were lost (i.e., bottom row in Fig 2B (losses)) or gained (i.e., left-most column in Fig 2B), the most obvious difference we observed was an excess of medium paths that were gained in burned sites compared to unburned sites. Very little of the movement redistribution was driven by changes in path thickness compared to complete gains or losses (S3 Fig), and path re-occurrences (however few) were primarily represented by medium and thick (but not thin) paths, to a greater degree in unburned sites (i.e., cells off the diagonal in Fig 2B and S3 Fig).

**Fig 3:**
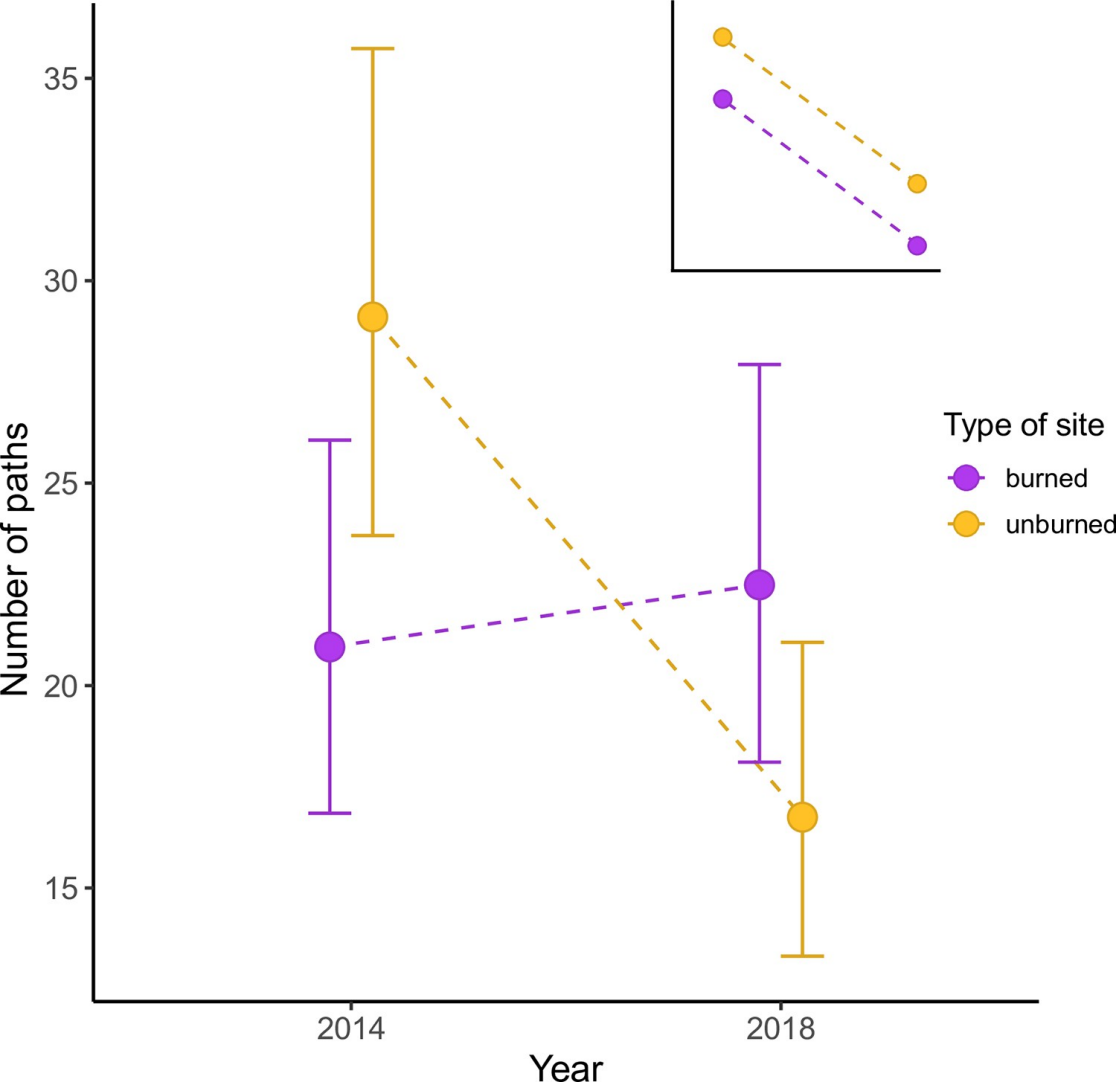
Overall number of deer paths in burned sites counteracted temporal trends observed in unburned sites. Given that our data unexpectedly showed a decrease in the number of paths over time in unburned ‘control’ sites (see Discussion for possible mechanisms), the inset shows expected change in path density between burned and unburned sites if wildfire had no influence on land use by deer (i.e., to help the reader develop a null expectation given these unexpected temporal trends). The data suggest that wildfire increased land use by deer relative to the unburned baseline. Points and error bars are fitted means and 95% confidence intervals from glmm, for burned (magenta points) or unburned (yellow points) areas.

**Fig 4:**
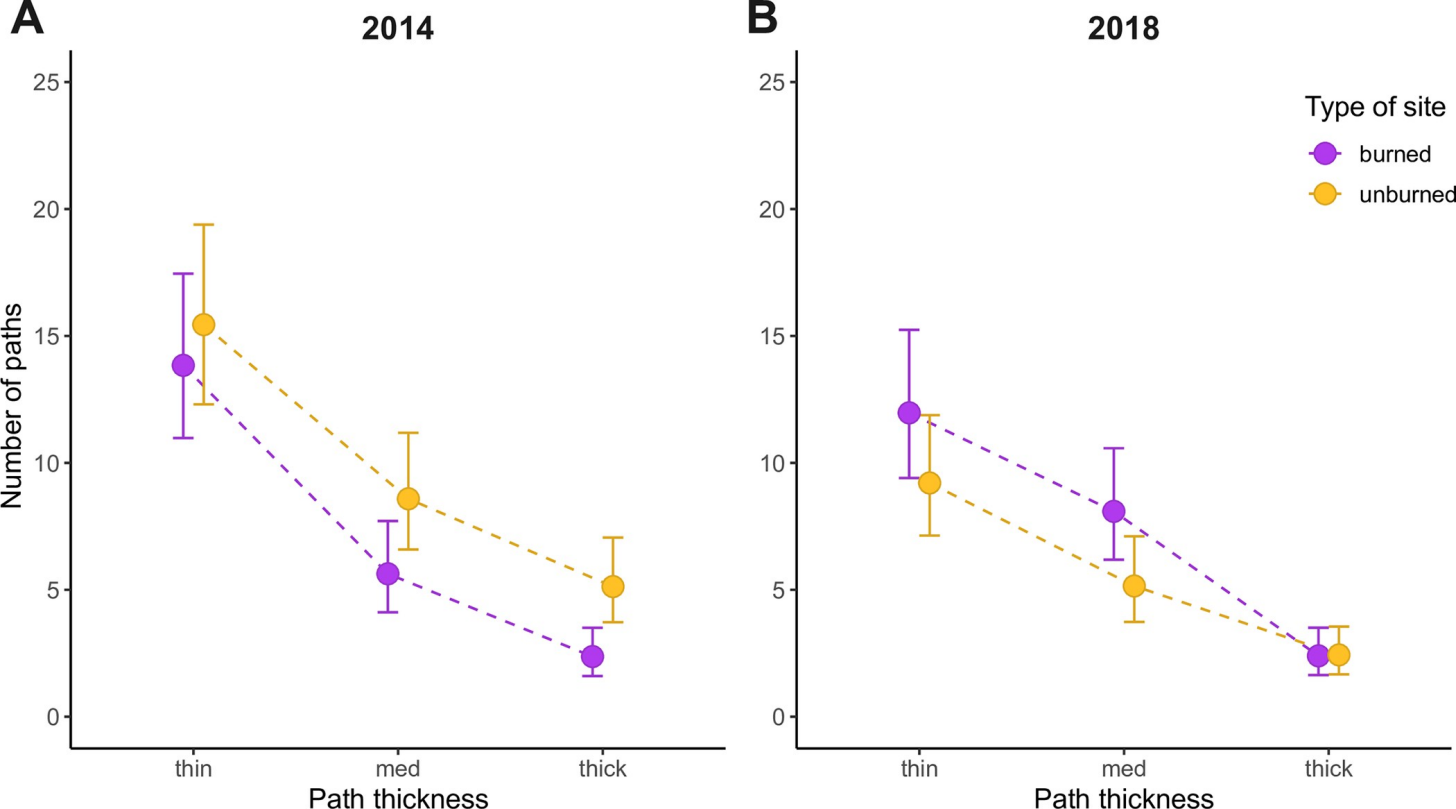
Reuse of paths within years (i.e., their thickness, a proxy for the directedness of movement) decreased in response to wildfire despite an increase in overall number of paths. Points and error bars are fitted means and 95% confidence intervals from glmm, for burned (magenta points) or unburned (yellow points) areas.

We also found support for our prediction that wildfire would increase the overall number of paths observed at a site (i.e., significant interaction between path density x plot type (*χ*^2^ = 18.5, *P* < 0.001); Fig 3)—this result was qualitatively equivalent when paths were weighted by thickness (*χ*^2^ = 18.0, *P* < 0.001; S5 Fig), which may be more reflective of overall activity as opposed to an increase in number of paths caused by each thick path being substituted by four thin ones (as an example). We specifically expected this increased number of paths to manifest through thin paths, as many thin paths would be expected if movement was less directed, but instead found that medium paths increased the most, compared to 2014 (pre-wildfire, our baseline; Fig 4). Regardless of burn history, deer paths were 0.77x less numerous in 2018 compared to 2014 regardless of wildfire (Fig 3), with possible explanations including climate differences, natural population cycles, or fire-caused mortality, and surprisingly, the greatest declines were observed in unburned sites (Fig 3). Sites fated to burn also exhibited less deer activity overall prior to the fires (i.e., a difference between points in 2014 in Fig 3), suggesting that these areas exhibited pre-existing differences in either number of deer or their movement behaviours. Although we can only speculate as to the cause of these pre-existing differences, one explanation stems the fact that unburnt sites had less shrub cover compared to sites fated to burn (S2 Fig; possibly explaining why the fire did not jump to these areas) resulting in lower perceived risk relative to sites fated to burn. Importantly, our BACI design (described in Methods) accounts for other sources of variation (e.g., climate, population cycles) to isolate the effects of wildfire, and strikingly, unburned sites in 2018 show similar path thickness distributions to sites in 2014, before any sites burned, as one would expect if the BACI design worked as intended.

## Discussion

We examined whether the reduction of habitat complexity by wildfire affected deer movement patterns, as one would expect if wildfire shifted the spatial structure of perceived risks vs. rewards in a landscape [4]. Our results suggest that, indeed, the risk/reward landscape is homogenized (i.e., decreased path fidelity within years), spatially restructured (i.e., decreased path fidelity between years, with the formation of new, moderately used paths), and is overall perceived as being less risky (i.e., increased overall activity, relative to unburned sites) in response to fire, compared to areas not impacted by fire. Although increased activity might also arise if animals explore and forge new routes in an unfamiliar environment, our surveys took place three years after the fire, enough time for animals to acclimate to their new surroundings. Note that these findings are consistent with both hypotheses (Fig 1A and 1B), rejecting the null hypothesis of no effect of fire on movement: that risk/reward still varies spatially but with a greater range of potential options of where paths can be formed. Interestingly, a recent study by Kreling et al. [40] that tracked 18 individual black-tailed deer (*Odocoileus hemionus columbianus*) in California using GPS collaring also showed that movement became more directed (i.e., measured by path tortuosity) and increased in activity (e.g., increased daily distances) overall in the first weeks following wildfire. Our study not only confirms these patterns but extends them to show that they persist 3 years post-fire and are prevalent enough to visibly affect where deer paths (an indicator of overall landscape use, aggregated across whole populations of deer as a collective behaviour) are formed. Below, we unpack several interesting and unexpected nuances and propose priorities for future research inspired by our results.

We detected hints of possible interdependencies between burned and unburned sites that raise interesting questions about the spatial scale of a wildfire’s influence, even in areas that were not directly impacted. An assumption of the BACI design is that treatments of interest (here, the presence or absence of recent wildfire) do not influence each other. Interestingly, we found a strong net loss of the overall number of paths in unburned sites that was not observed in burned sites. This net loss may have an interesting explanation: that deer were being drawn from unburned areas into burned ones, a phenomenon sometimes referred to as the “magnet effect”. Magnet effects have been observed for deer following fires [41, 42], potentially due to the combination of reduced risks and increases in high-quality forage due to nitrogen release by low-intensity fires [43, 44]—however, several studies [40, 45, 46] also found evidence that deer do not shift their home ranges entirely to use burned areas only, instead expanding their home ranges to include burned areas. This finding suggests that unburned sites may be perceived as offering unique benefits [37], such as shelter from the elements or familiar surroundings, which may come with opportunity costs when environments change [40, 46]. If this is true, we hypothesize that risks and rewards are being assessed at different spatial scales [47]: risks based on vegetation cover (or its absence) in the immediate vicinity (i.e., the scale of a subplot) vs. rewards being accessed via transiting between burned and unburned locations. Magnet effects are only possible if fire occurs on a finer spatial grain than scales of individual movement [25], as is true in our study where mule deer are our primary path formers. An important caution, however, is that the patterns we observed are consistent with but cannot be conclusively attributed to magnet effects without additional data on how rewards are distributed, which cannot be inferred given the spatial and temporal resolution of our satellite imagery.

Although the magnet effect would explain relative changes in path density between burned and unburned sites, it would not explain why movement activity was overall lower in 2018 compared to 2014 in our study (i.e., when aggregating across all sites; Fig 4). This observation might simply reflect natural background cycles in deer population sizes or movement dynamics unrelated to the fire season, or, may reflect fire-caused mortalities (either during the fire or via starvation afterwards). For example, Kreling et al. [40] found that deer activity increased in the weeks following a wildfire, potentially in an attempt to combat resource scarcity following a fire. Indeed, although public estimates of deer abundances are taken at a spatial scale that extends far beyond the reserve, deer populations in Hunt Zone A (the zone encompassing the reserve) dropped during (130,266 individuals) and in the couple years following the 2015 fires (i.e., 97,520 individuals in 2017), compared to levels before the fires in 2014 (~162,000 individuals; https://wildlife.ca.gov/Conservation/Mammals/Deer/Population#32712445-population-by-hunt-zone). We caution, however, that we cannot use these data to say for certain whether these population trends applied to the reserve or whether they were caused by wildfires. Testing the scale of a wildfire’s influence could be achieved by comparing regions (e.g., replication at the scale of all of McLaughlin Reserve) that either received no wildfire at all vs. partial wildfire, and observing if unburned sites varied through time more strongly in regions impacted by wildfire.

Our hypothesis for how habitat structure would affect the risk environment was specific to large grazers, but how might our findings apply to other prey species? Whether risks and rewards decrease or increase overall with a loss of habitat structure likely depends on context (see review by Wirsing [6]), namely, the interaction between characteristics of the prey (e.g., ability to detect predators, escape or defence behaviours [48–50], their predators (e.g., hunting tactics [6, 51]), and how the loss of complexity was achieved [32]. Many prey species, for example, small-bodied species with faster, larger, or aerial predators, require habitat structure in order to hide. A loss of structure may limit hiding opportunities, exposing prey to predators and triggering predator avoidance behaviours, such as reduced movement [9] and reduced foraging [52]. Indeed, many studies show that predators of small mammals gravitate towards burned or burning habitat (see review by Nimmo et al. [25]), as reduced cover exposes prey for more efficient capture (see study by Leahy et al. [53])—however, decreased habitat complexity might alternatively reduce the hunting efficiency of some predators, such as Harrier hawks, who hunt from tall perches provided by woody plants [32]. In our study system specifically, we would expect the change in vulnerability of deer to predators in response to wildfire to be strongest for stalking predators, like mountain lions, compared to predators like coyotes that chase prey at longer distances that may be advantaged by reduced obstacles. No synthesis to our knowledge has compared how and why disturbances affect the risk environment differently depending on characteristics of prey (e.g., body size) and their predators (e.g., hunting strategies).

An increasing interest in ecology is to understand how animals base their movements on spatial information [18, 19, 54, 55], given that organismal movement connects subunits in ecosystems [56]. The ability for animals to make different, beneficial decisions as conditions change may be crucial for survival as environments change [57, 58], both gradually or acutely (as is the case with widespread wildfire). We show that deer exhibit high behavioural plasticity in how they transit through a landscape within their home range among years in ways that are exaggerated in areas impacted by recent wildfire—this contrasts the high degree of inflexibility observed in ungulates in shifting their home ranges (e.g., [37, 40]). Further, despite this high plasticity, there remains marked directionality to movement (i.e., an increase in medium paths, relative to small and large paths) in ways that might reflect collective decision-making as good quality paths (i.e., those suitably balancing risk vs. reward) are reinforced and poor quality ones are left behind, forming a roadmap for deer in landscape [10]. Our results also emphasize the scale dependence of spatial information important to movement decisions. For example, fire may reduce landscape complexity from the perspective of individual animals experiencing a given location on a landscape while simultaneously enhancing landscape complexity when aggregated across all locations (including a mix of fire histories) an individual experiences as it moves over a given period of time.

In conclusion, our study provides directions for future research on how the effects of disturbances such as wildfire are distributed among species and spatial scales, with potential for indirect consequences for other species (e.g., predators and prey of species impacted by disturbance). Beyond these future directions, we highlight the intersection of this research and two current global issues. First, we present this study as an example of scientific innovation and dedication to discovery in response to extraordinary research disruption (i.e., the COVID-19 pandemic). Second, extreme and unpredictable events have dramatically increased in frequency and intensity in recent years [59]. In response, more and more studies, like ours, opportunistically seek to leverage these events as natural experiments to document biological consequences (e.g., impacts of a heatwave on intertidal communities [60]). Given that these experiments are unplanned and that extreme events are unpredictable but not necessarily rare, we advocate for strategic long-term monitoring of ecological systems in order to devise a priori objectives and allow for an expanded scope of inferences. Our study underscores the importance of including animal movement in long-term monitoring programs (e.g., with radio collars) especially given that behavioural changes have been proposed [61] as the first early-warning sign for collapse (and inversely, for recovery [62]) of wildlife populations.

## Supporting information

S1 Fig Study area at McLaughlin Natural Reserve. Study area at McLaughlin Natural Reserve showing unburned vs. burned sites as yellow vs. purple map pins respectively (a), and a burned site before (left) vs. after (right) wildfire (b). The 2015 Rocky and Jerusalem fires burned ~half of the landscape, allowing comparisons. Areas untouched by the fires are shown on the map in greyscale. Map data in (a) are from the USGS National Map Viewer, photos in (b) taken by R. Germain.
(PNG)

S2 Fig Impacts of wildfire on shrub cover. A comparison of shrub cover in plots (as a percentage of total area) in 2014 vs. 2018, in plots that either were (i.e., ‘burned’, purple points) or were not (i.e., ‘unburned’, yellow points) impacted by the 2015 wildfires.
(PDF)

S3 Fig Example of path fidelity within and between years. Contingency table (A) counts the number of path appearances in 2018, path disappearances from 2014, and thickness transitions for recurring paths between both years for a hypothetical site (B). The “None” categories represent an absence of a path in the respective year. For example, the table shows that (1) three thin paths disappeared by 2018, (2) one new medium path was created by 2018, (3) one medium path was shorter in 2018 but did not change in thickness, and (4) one medium path became thicker in 2018.
(PDF)

S4 Fig Heat map associated with Fig 2B, except separating out burned and unburned sites. Counts are presented on their raw (as opposed to proportional, as in Fig 2B) scale.
(PNG)

S5 Fig Total use of paths within years when (A) without (i.e., raw # of paths) and (B) with weighting by path thickness. Results of panel (A) are presented in the main manuscript. Points and error bars are fitted means and 95% confidence intervals from glmm, for burned (magenta points) or unburned (yellow points) areas.
(PDF)

S1 File Impacts of fire on cover habitat. (DOCX)
