# Supplementary figures and images for: Landscape use by large grazers in a grassland is restructured by wildfire
Source: PLoS One. 2024 Feb 13;19(2):e0297290. doi: 10.1371/journal.pone.0297290 (PMC10863880; doi:10.1371/journal.pone.0297290)

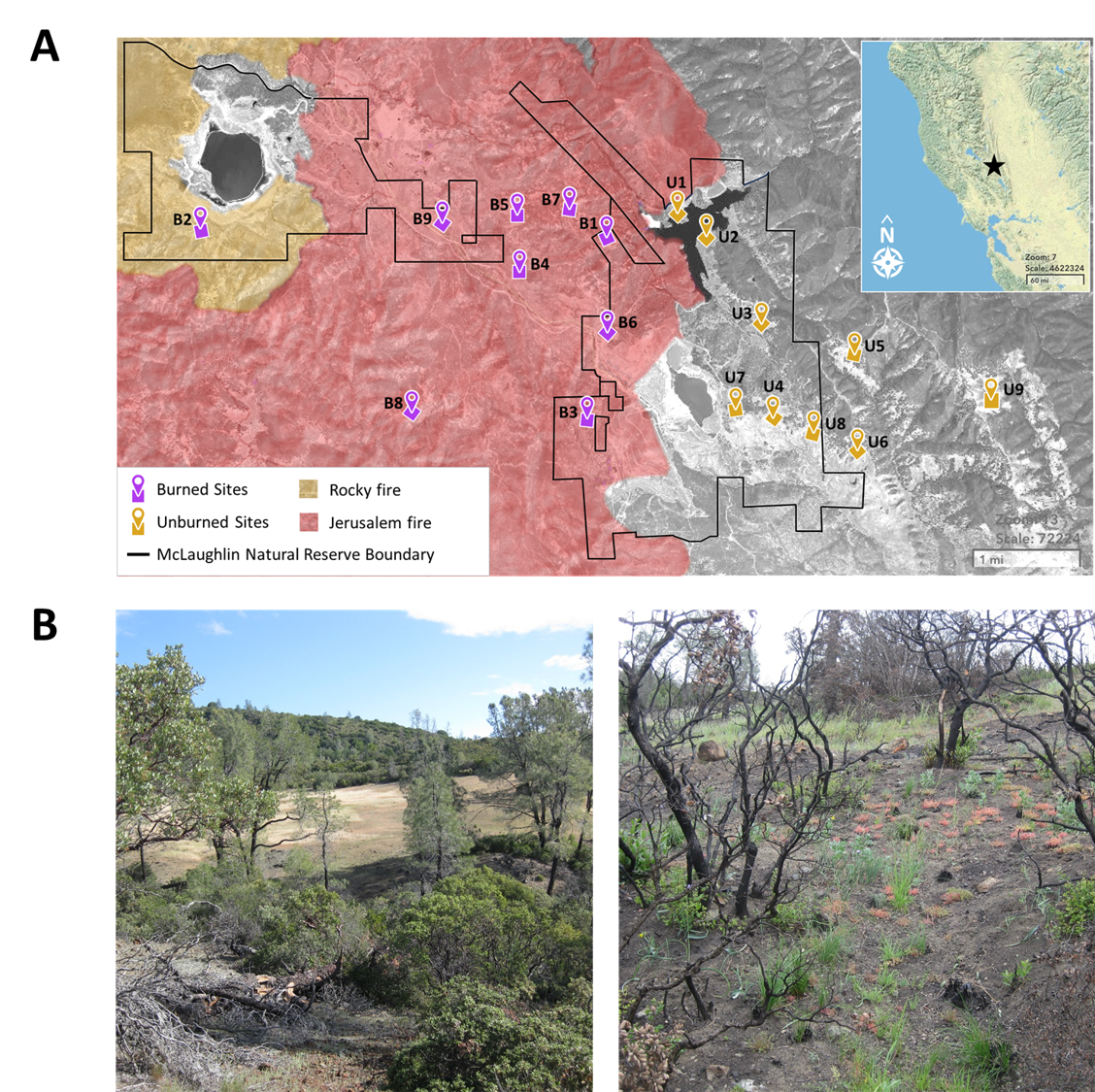

Supplement: S1 Fig — Study area at McLaughlin Natural Reserve showing unburned vs. burned sites as yellow vs. purple map pins respectively (a), and a burned site before (left) vs. after (right) wildfire (b). The 2015 Rocky and Jerusalem fires burned ~half of the landscape, allowing comparisons. Areas untouched by the fires are shown on the map in greyscale. Map data in (a) are from the USGS National Map Viewer, photos in (b) taken by R. Germain. (PNG) [file pone.0297290.s001.png]

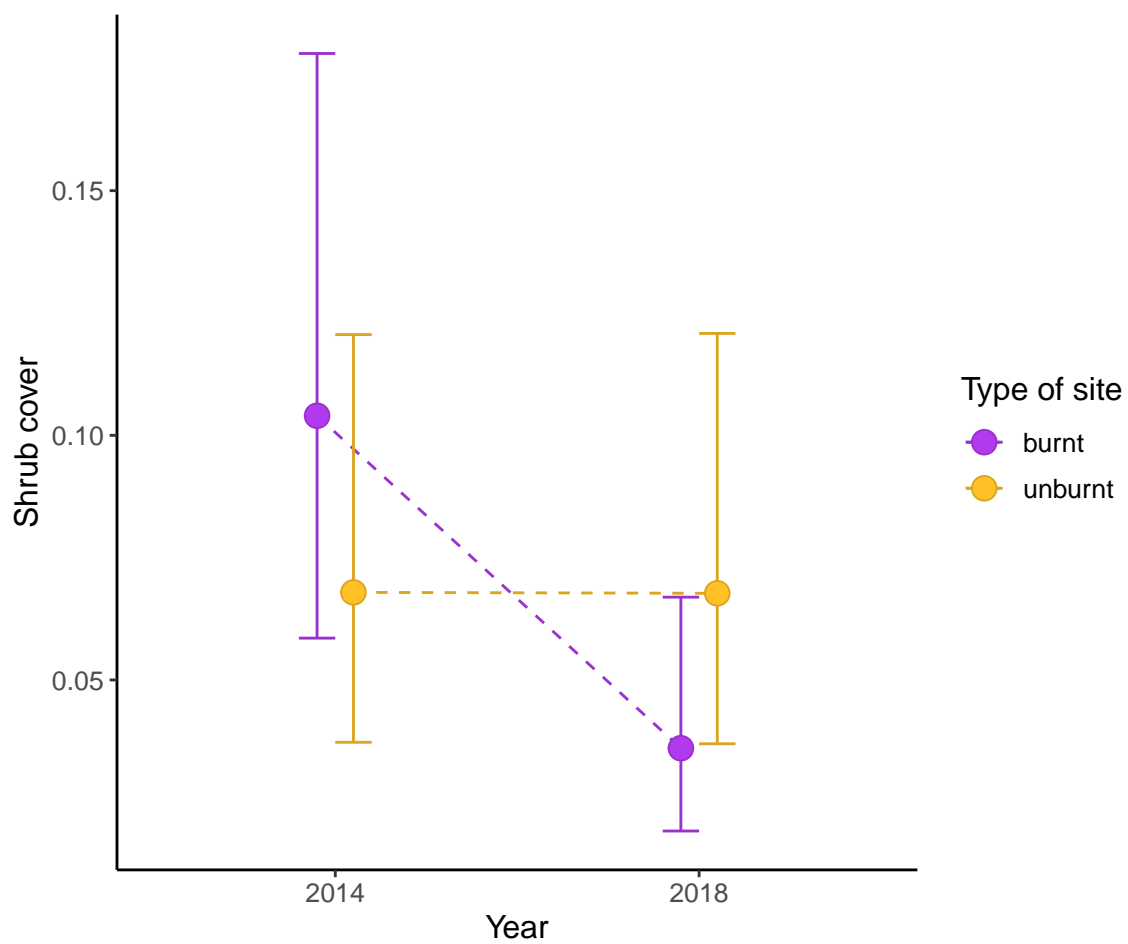

Supplement: S2 Fig — A comparison of shrub cover in plots (as a percentage of total area) in 2014 vs. 2018, in plots that either were (i.e., ‘burned’, purple points) or were not (i.e., ‘unburned’, yellow points) impacted by the 2015 wildfires. (PDF) [file pone.0297290.s002.pdf]

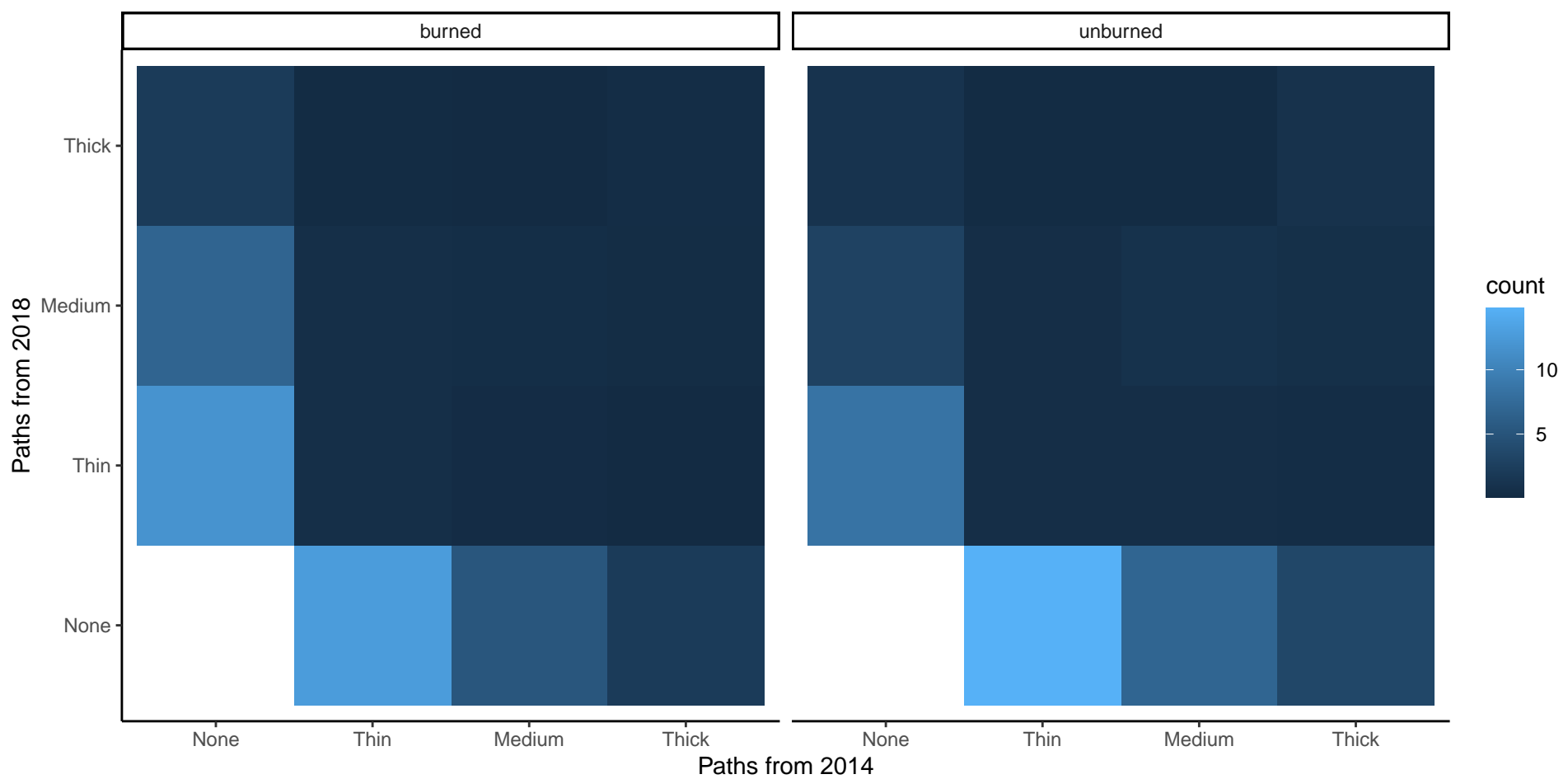

Supplement: S3 Fig — Contingency table (A) counts the number of path appearances in 2018, path disappearances from 2014, and thickness transitions for recurring paths between both years for a hypothetical site (B). The “None” categories represent an absence of a path in the respective year. For example, the table shows that (1) three thin paths disappeared by 2018, (2) one new medium path was created by 2018, (3) one medium path was shorter in 2018 but did not change in thickness, and (4) one medium path became thicker in 2018. (PDF) [file pone.0297290.s003.pdf]

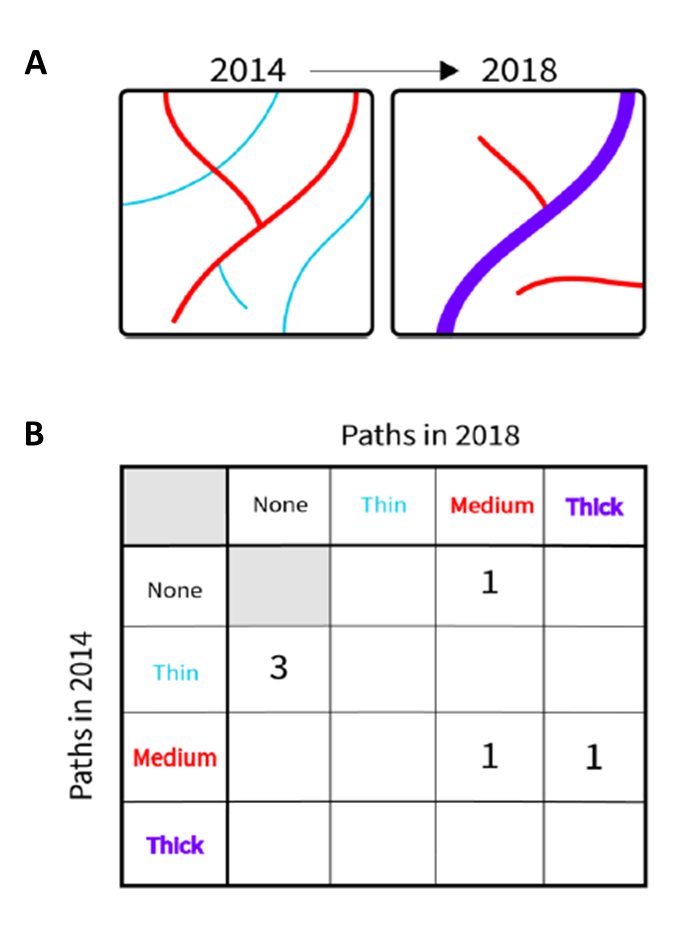

Supplement: S4 Fig — Counts are presented on their raw (as opposed to proportional, as in Fig 2B) scale. (PNG) [file pone.0297290.s004.png]

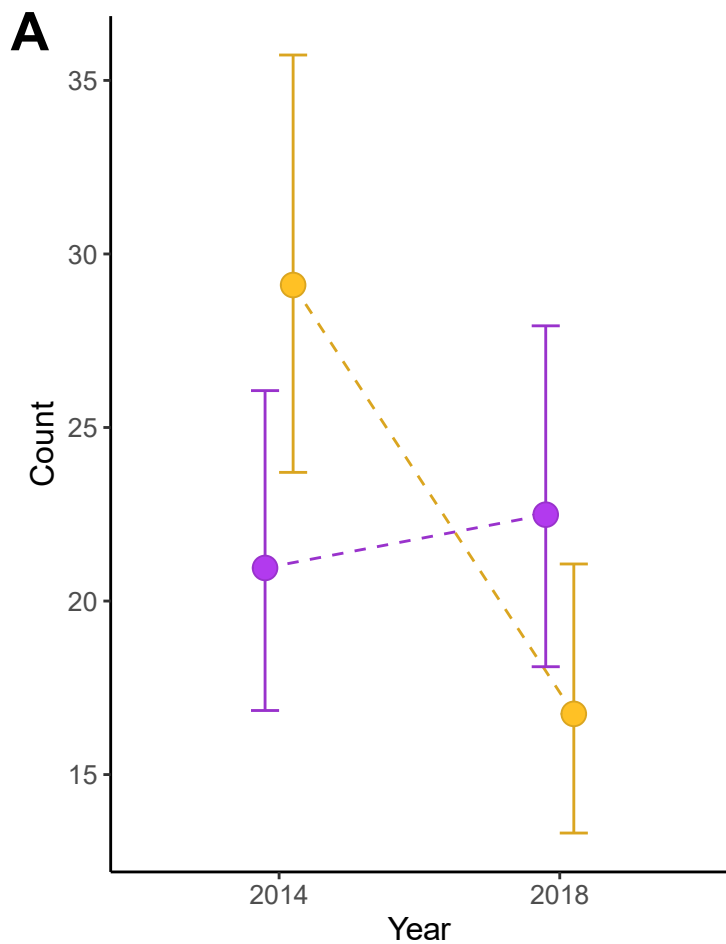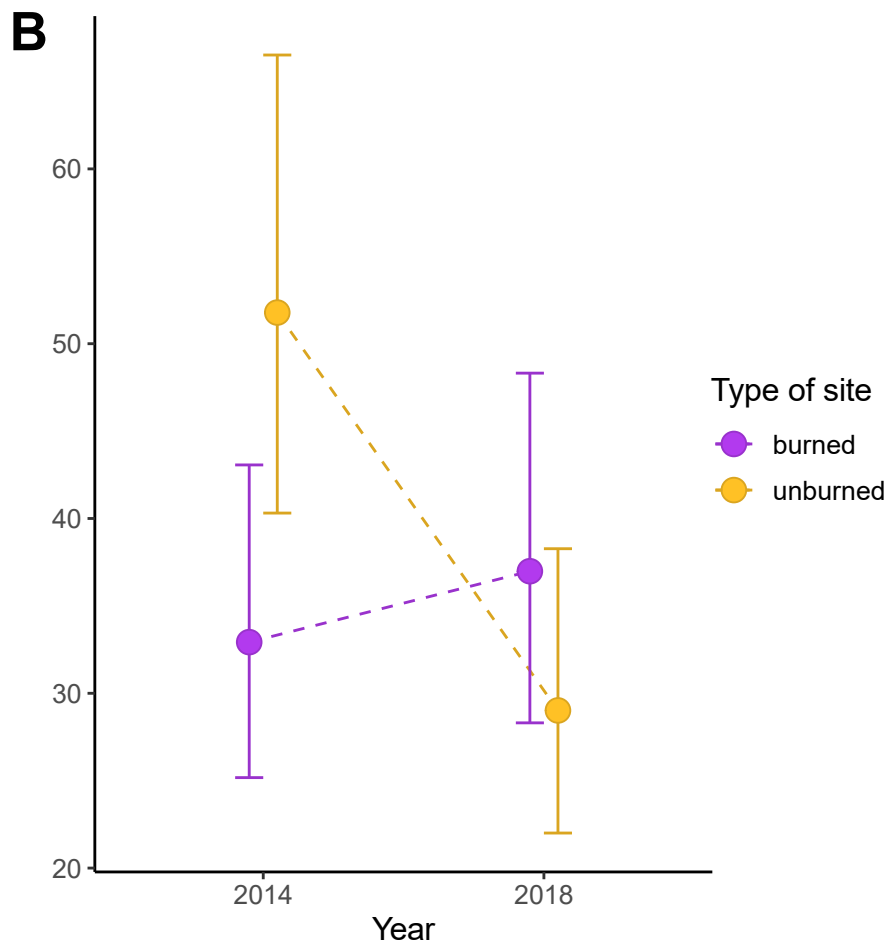

Supplement: S5 Fig — Total use of paths within years when (A) without (i.e., raw # of paths) and (B) with weighting by path thickness. Results of panel (A) are presented in the main manuscript. Points and error bars are fitted means and 95% confidence intervals from glmm, for burned (magenta points) or unburned (yellow points) areas. (PDF) [file pone.0297290.s005.pdf]
