# Supplementary material for: Landscape use by large grazers in a grassland is restructured by wildfire
Source: PLoS One. 2024 Feb 13;19(2):e0297290. doi: 10.1371/journal.pone.0297290 (PMC10863880; doi:10.1371/journal.pone.0297290)
Supplement: S1 File — (DOCX) [file pone.0297290.s006.docx]

**Supplementary Methods**

***Impacts of fire on cover habitat***

We hypothesized that animal movement would be affected by fire due to removal of cover habitat (i.e., tall shrubs, trees). To test how strongly fire impacted the cover environment, and to decouple these impacts from temporal changes in cover over time, we measured % cover in every plot in 2014 and 2018 using the same remotely sensed image data used to extract animal paths. To estimate % cover data from images, we used ImageJ v. 1.53. On each image, we adjusted the color threshold settings until all pixels containing living shrub and tree cover were highlighted. We then used ‘particle analysis’ to quantify the proportion of pixels in each image that were highlighted. We needed to readjust the color threshold settings for each image due to differences in image lighting.

***Stats.*** We used a generalized linear mixed effects model with % cover (as a proportion, for analysis) as the response variable, using a ‘beta’ error distribution as is most appropriate for proportion data. Fixed effects included year, plot type (impacted or not impacted by fire), and their interaction, with ‘plot’ included as a random factor to account for resampling of each plot in two years. We also repeated our analysis except testing differences in cover among plots that would or would not eventually burn, in 2014 only, before the wildfires passed through. A significant difference between plot types might help explain why some plots burned (i.e., ones with more cover) and others did not. As with our main analyses, we used R package ‘DHARMa’ to confirm that our statistical models met all assumptions.

***Results and Discussion.*** As predicted, cover was significantly reduced in burned sites only, with 63% losses on average, compared to a non-significant 5% gain in cover among years for unburned sites (i.e., significant interaction between path fidelity x plot type interaction (*χ*^2^ = 35.4, *P* < 0.001); S2 Fig). Prior to fire, in 2014, sites that would or would not eventually burn did not significantly differ in cover (i.e., non-significant effect of plot type (*χ*^2^ = 1.1 *P* = 0.285); S2 Fig).

**
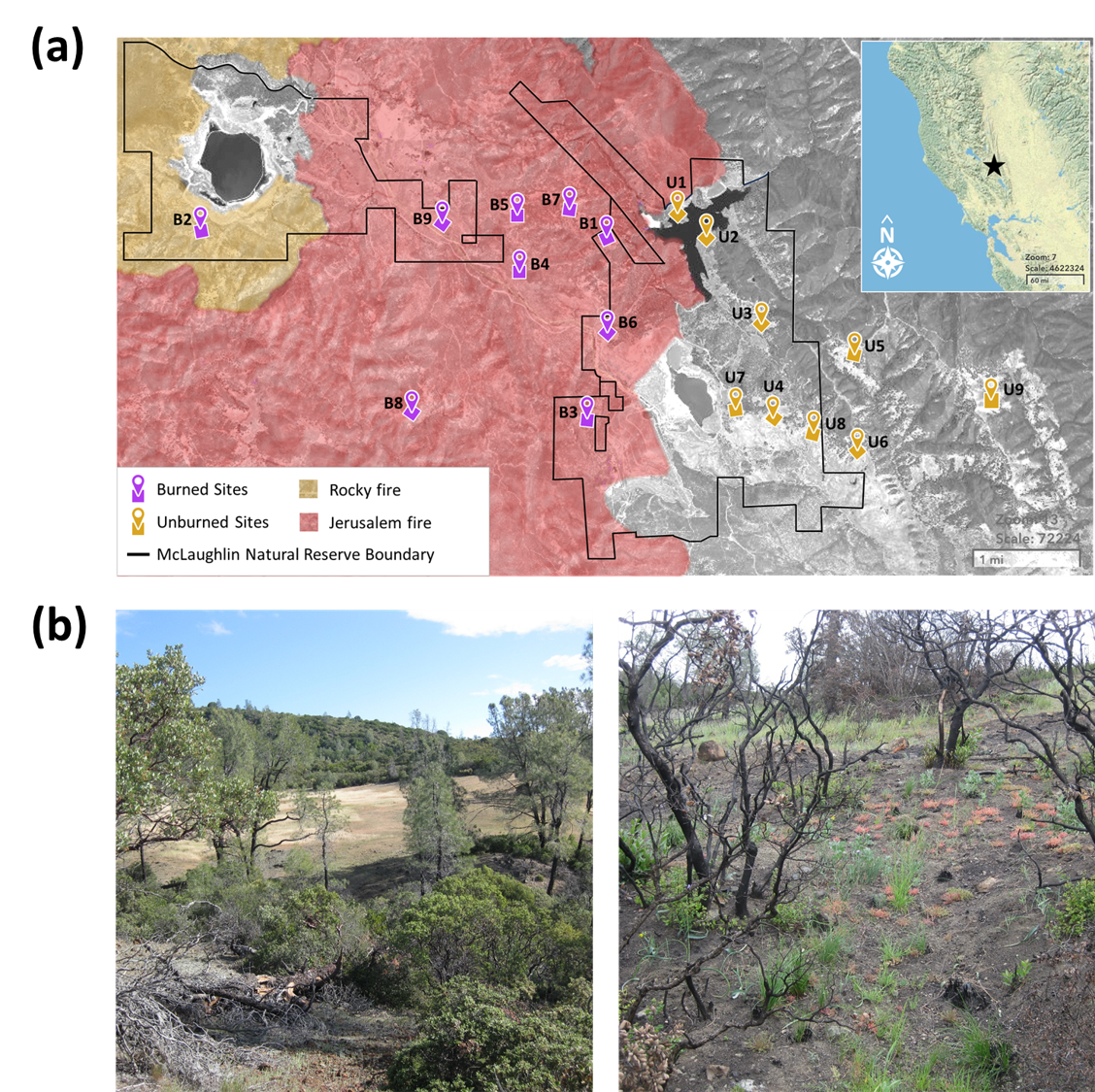
**

**Fig. S1** Study area at McLaughlin Natural Reserve showing unburned vs. burned sites as yellow vs. purple map pins respectively (a), and a burned site before (left) vs. after (right) wildfire (b). The 2015 Rocky and Jerusalem fires burned ~half of the landscape, allowing comparisons. Areas untouched by the fires are shown on the map in greyscale. Map data in (a) are from the USGS National Map Viewer, photos in (b) taken by R. Germain.


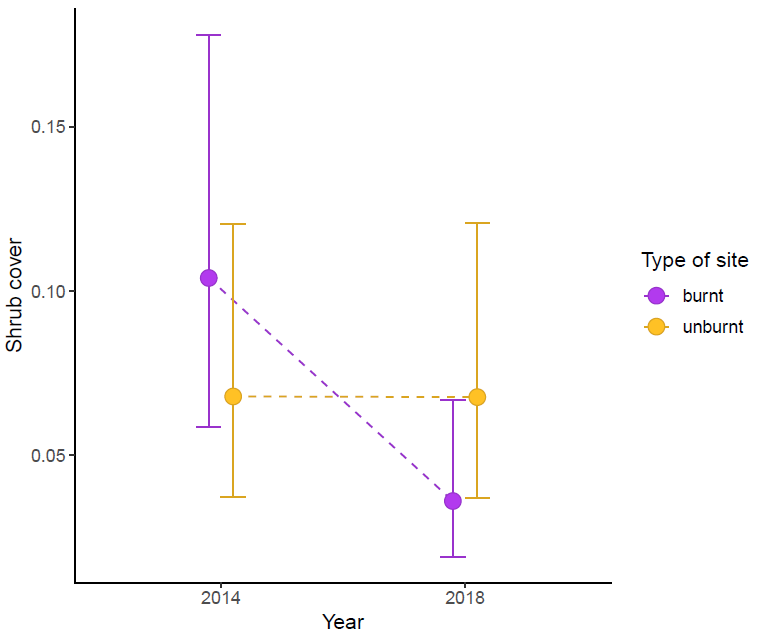


**Fig. S2** A comparison of shrub cover in plots (as a percentage of total area) in 2014 vs. 2018, in plots that either were (i.e., ‘burned’, purple points) or were not (i.e., ‘unburned’, yellow points) impacted by the 2015 wildfires.


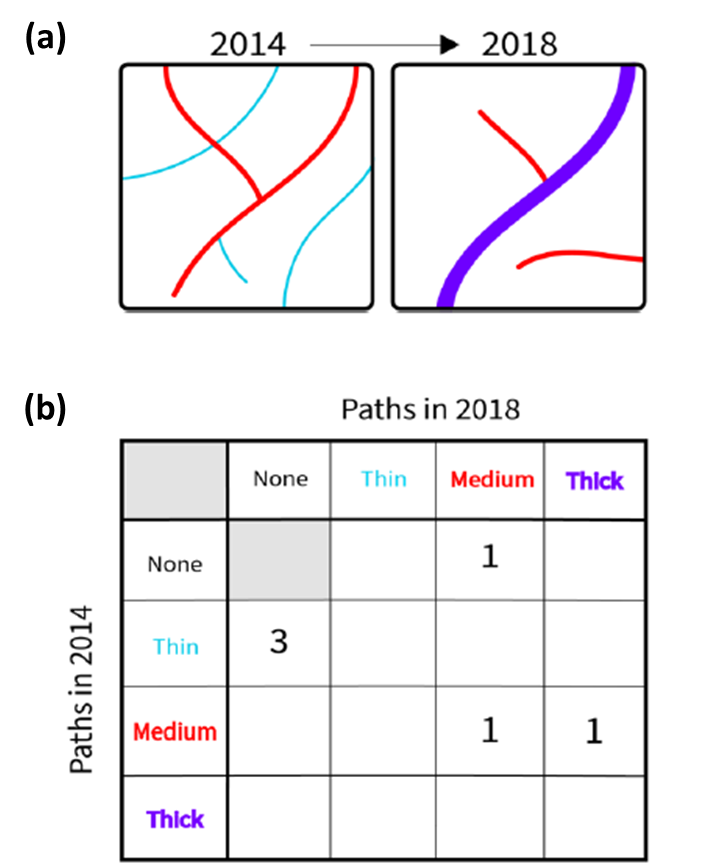


**Fig. S3** Example of path fidelity within and between years. Contingency table (a) counts the number of path appearances in 2018, path disappearances from 2014, and thickness transitions for recurring paths between both years for a hypothetical site (b). The “None” categories represent an absence of a path in the respective year. For example, the table shows that (1) three thin paths disappeared by 2018, (2) one new medium path was created by 2018, (3) one medium path was shorter in 2018 but did not change in thickness, and (4) one medium path became thicker in 2018.


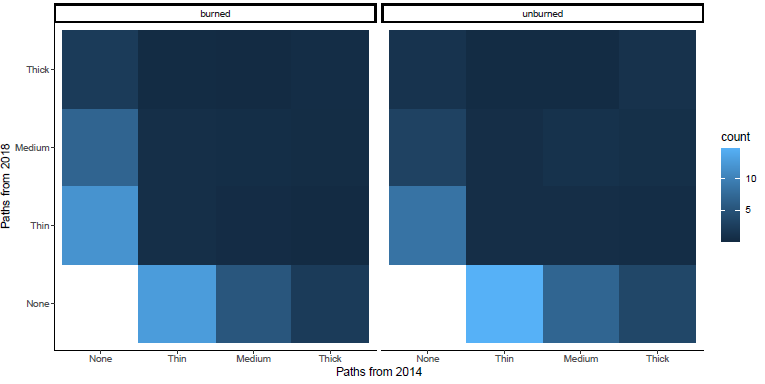


**Fig. S4** Heat map associated with Figure 2b, except separating out burned and unburned sites, showing them on their raw (as opposed to proportional, as in Figure 2b) scale.
